# The role of TET2-mediated *ROBO4* hypomethylation in the development of diabetic retinopathy

**DOI:** 10.1186/s12967-023-04310-4

**Published:** 2023-07-10

**Authors:** Liangliang Zhao, Haitao Xu, Xin Liu, Yan Cheng, Jia’nan Xie

**Affiliations:** Department of Ophthalmology, The Second Hospital of Jilin University, Changchun, Jilin China

**Keywords:** Diabetic retinopathy, Hypomethylation, Retinal microangiopathy, ROBO4, TET2

## Abstract

**Background:**

In diabetic retinopathy, increasing evidence points to a link between the pathogenesis of retinal microangiopathy and the endothelial cell-specific factor roundabout4 (ROBO4). According to earlier research, specificity protein 1 (SP1) enhances the binding to the *ROBO4* promoter, increasing Robo4 expression and hastening the progression of diabetic retinopathy. To determine if this is related to aberrant epigenetic modifications of *ROBO4*, we examined the methylation level of the *ROBO4* promoter and the corresponding regulatory mechanism during the course of diabetic retinopathy and explored the effect of this mechanism on retinal vascular leakage and neovascularization.

**Methods:**

The methylation level of CpG sites in the *ROBO4* promoter was detected in human retinal endothelial cells (HRECs) cultured under hyperglycemic conditions and retinas from streptozotocin-induced diabetic mice. The effects of hyperglycemia on DNA methyltransferase 1, Tet methylcytosine dioxygenase 2 (TET2), 5-methylcytosine, 5-hydroxymethylcytosine, and the binding of TET2 and SP1 to the *ROBO4* promoter, as well as the expression of ROBO4, zonula occludens 1 (ZO-1) and occludin were examined. Short hairpin RNA was used to suppress the expression of TET2 or ROBO4 and the structural and functional changes in the retinal microvascular system were assessed.

**Results:**

In HRECs cultured under hyperglycemic conditions, the *ROBO4* promoter methylation level decreased. Hyperglycemia-induced TET2 overexpression caused active demethylation of *ROBO4* by oxidizing 5-methylcytosine to 5-hydroxymethylcytosine, which enhanced the binding of SP1 to *ROBO4*, increased the expression of ROBO4, and decreased the expression of ZO-1 and occludin, leading to the abnormalities in monolayer permeability, migratory ability and angiogenesis of HRECs. The above pathway was also demonstrated in the retinas of diabetic mice, which caused leakage from retinal capillaries and neovascularization. Inhibition of TET2 or ROBO4 expression significantly ameliorated the dysfunction of HRECs and retinal vascular abnormalities.

**Conclusions:**

In diabetes, TET2 can regulate the expression of ROBO4 and its downstream proteins by mediating active demethylation of the *ROBO4* promoter, which accelerates the development of retinal vasculopathy. These findings suggest that TET2-induced *ROBO4* hypomethylation is a potential therapeutic target, and anti- TET2/ROBO4 therapy is anticipated to emerge as a novel strategy for early intervention and delayed progression of diabetic retinopathy.

## Background

Diabetic retinopathy (DR) is a serious microvascular complication of diabetes mellitus and is the leading cause of blindness in working-age populations worldwide [1, 2]. In 2020, there were 103.12 million adults with DR, and this number was projected to rise to 160.5 million by 2045 [3]. Pathological changes divide DR into two stages: non-proliferative diabetic retinopathy (NPDR) and proliferative diabetic retinopathy (PDR) [4]. The NPDR stage is characterized by damage or loss of endothelial cells, pericyte apoptosis, relaxation of tight junctions, and increased permeability, which leads to destruction of the blood–retinal barrier [5, 6]. During the proliferative stage, extensive capillary occlusion causes severe local retinal hypoxia, resulting in upregulation of numerous growth factors and inflammatory mediators, which leads to retinal neovascularization [7, 8]. Therefore, protecting the integrity of retinal microvessels and inhibit retinal neovascularization is important to prevent and treat DR.

Roundabout4 (ROBO4), a cell surface protein expressed specifically in endothelial cells, is involved in regulating endothelial cell permeability and angiogenesis [9, 10]. In recent 10 years, accumulated evidence suggests that Robo4 expression is closely related to the occurrence and development of DR. ROBO4 expression was elevated in the fibrovascular membranes of PDR patients [11], in the retinas of streptozotocin-induced diabetic rats, and in REP cells cultured under hyperglycemic or hypoxic conditions [12, 13]. Moreover, microRNA-411 and microRNA-15a play protective roles, ameliorating DR progression, by inhibiting ROBO4 and vascular endothelial growth factor (VEGF) expression [12, 14]. MicroRNA-146a-5p and microRNA-125b-5p regulate ROBO4 expression by targeting hypoxia-inducible factor-1α and specificity protein 1 (SP1) during DR development [13]. In our previous study, we found that SP1 is a transcription factor that regulates the transcription and expression of *ROBO4* in human retinal endothelial cells (HRECs). Under hyperglycemic stimulation, SP1 expression increased, and its binding to the *ROBO4* promoter was enhanced, which led to abnormal migration, permeability, and angiogenesis of HRECs. Inhibition of the SP1/ROBO4 pathway significantly ameliorated hyperglycemia-induced HRECs dysfunction [15]. However, the mechanism regulating binding of SP1 to *ROBO4* remains unclear, although it is suspected to be related to the methylation level of *ROBO4* promoter.

DNA methylation is an important form of epigenetic modification and has been widely studied in the pathogenesis of DR over the past decade. In diabetes, DNA methyltransferase (DNMT) expression is activated, and mitochondrial DNA is hypermethylated in retinal mitochondria, which impairs the transcription of genes encoded by this DNA [16]. Ten–eleven translocation dioxygenases (TETs) are also activated, which can rapidly hydroxymethylate 5-methylcytosine (5mC) to 5-hydroxymethylcytosine (5hmC) [17] and activate the transcription of matrix metalloproteinase-9 (MMP-9) and ras-related C3 botulinum toxin substrate 1, leading to oxidative stress and mitochondrial damage [18, 19]. Some inflammatory-related cytokines, such as tumor necrosis factor and NOD-like receptor protein 3, are increased in serum, and the DNA methylation levels of their promoters are decreased in DR patients, triggering the inflammatory reaction of DR [20, 21]. In studies on the mechanism of pathological neovascularization in DR, maternally expressed gene 3 (MEG3) was found to be a protective factor that inhibited VEGF expression and suppressed endothelial–mesenchymal transition [22, 23]. Under hyperglycemic conditions, *MEG3* promoter hypermethylation reduced MEG3 expression, leading to pathologic migration and neovascularization in HRECs [24]. These studies suggested that aberrant DNA methylation is closely involved in the pathological process of DR. Therefore, it can be inferred that hyperglycemic stimulation may alter methylation modification of the *ROBO4* promoter and regulate ROBO4 expression in the process of DR development.

In this study, the methylation level of the *ROBO4* promoter was examined in HRECs and retinas from diabetic mice. We further investigated the mechanism of aberrant methylation and its effect on the expression of ROBO4 and the tight junction proteins zonulae occludente 1 (ZO-1) and occludin, and validated the role of this pathway in the progression of retinal microangiopathy in DR.

## Materials and methods

### Cells

HRECs (ScienCell, Carlsbad, CA, USA) were cultured in endothelial cell medium containing 10% fetal bovine serum, 1% endothelial cell growth supplement, 1% penicillin/streptomycin solution, and incubated at 37 °C in a humidified atmosphere with 5% CO_2_. Cells were harvested for experiments after 4–5 generations. Cells were treated with a high concentration of glucose (25 mmol/l) to simulate hyperglycemic conditions, or with normal glucose (5.5 mmol/l) concentrations, as the control group. HRECs were transfected with a lentiviral expression vector containing *TET2* short-hairpin RNA (shRNA) or control shRNA gene for 48 h at a multiplicity of infection of 60. Following transfection, cells were treated with glucose as described above for 7 days and then collected for further analysis.

### Animals

Six- to eight-weeks-old C57BL/6 mice were reared under specific pathogen-free conditions at a temperature of 22 ± 1 °C and a humidity of 45–55%, with a 12-h light/dark cycle. After a week of adaptive feeding, a diabetic mouse model was induced by intraperitoneal injection of streptozotocin (60 mg/kg) daily, for 5 consecutive days, and 7 days after the last injection, the fasting glucose levels in the tail vein blood of the mice were measured using a glucose test strip. Mice with blood glucose over 11.1 mmol/l were considered diabetic. Information on blood glucose level and body weight for the validation of diabetes establishment was provided in Table 1. These mice received intravitreal injections of adeno-associated virus containing *TET2* shRNA, *ROBO4* shRNA, or control shRNA gene, which were generated and packaged by Wanlei Biotechnology (Shenyang, China). Intravitreal injection was performed as described [25], briefly, the superior temporal quadrant of the sclera was exposed under the operating microscope, and the sclera was perforated with a 1 ml syringe needle at 1-mm posterior to the limbus. A microsyringe was used to obtain 1 μl of vitreous through the perforation, at an angle of 45° in the direction of the optic nerve, after which 1 μl of adenovirus with a titer of 1 × 10^10^ PFU/ml was injected. The needle remained in situ for 30 s post-injection and was then removed rapidly. The adenovirus was re-injected every 4 weeks. The infection rate was measured 72 h after the first injection. Mice in each group were kept for 10 weeks (thus, two virus injections) or 20 weeks (thus, four virus injections), euthanized by cervical dislocation, and retinal tissues were collected immediately. Some tissues were frozen in liquid nitrogen and stored at −80 °C, while others were fixed with 4% paraformaldehyde for further use.

**Table 1:** Basic characteristics of STZ-induced diabetic mice

Mouse group	n	Body weight (g)	Blood glucose (mmol/l)
NC	12	18.28 ± 1.20	6.83 ± 1.77
DM	50	16.54 ± 1.95*	13.96 ± 2.43*

Data represent the mean ± standard deviation

*NC* normal mice, *DM* diabetic model mice

^*^p < 0.05 vs NC

### Bisulfite sequencing polymerase chain reaction

Genomic DNA was extracted from HRECs exposed to normal or hyperglycemic conditions and retinas of wild-type or diabetic mice, using a tissue/cell genome DNA isolation kit (Bioteke Corporation, Beijing, China). Genomic DNA was modified with bisulfite using an EZ DNA Methylation-Gold kit (Zymo Research, Irvine, CA, USA). Based on the methylation sites in the *ROBO4* promoter region, three primer pairs were designed (see Table 2). Polymerase chain reaction (PCR) amplification was performed, and the resulting amplicons were cloned into a TA vector. The methylation state of the targeted sequences was analyzed using DNA sequencing.

**Table 2:** Oligonucleotide primer sets for BSP

Primer no	Primer sequence	Size
1	F: GGGTGAGGTTTTGTTGAAGGTGATTTT R: ACATTAATTTTAAAAACACTAATTAAATAC	299
2	F: GTAGATTGTTGATAGTGATATTTTTGATAAGTTG R: CCACTAACATCCAATACCTAATACATTAT	302
3	F: TTAGGGGTGTAGAAAGGTATAATTTAAA R: ATAAAACCTCCAAATACATCCAAAC	236

*F* forward primer, *R* reverse primer

### Real-time quantitative PCR

Total RNA was extracted from HRECs or retinal tissues using TRIzol reagent (Bioteke), and the purity and concentration were measured using a NanoDrop 2000 Spectrophotometer (Thermo Fisher Scientific, Waltham, MA, USA). After producing cDNA real-time quantitative PCR (RT-qPCR) was performed using an Exicycler 96 (Bioneer, Daejeon, Korea), with normalization to β-actin levels. The *ROBO4*, *DNMT1*, and *TET2* primers, procured from Genscript Biotech Corporation (Nanjing, China), are listed in Table 3. The cycle threshold (Ct) values were calculated and fold-changes were calculated using the ΔΔCt method.

**Table 3:** Primer sequences of target genes in real-time quantitative PCR

Gene^a^	Primer sequence^b^
Hs *β-actin*	F: GGCACCCAGCACAATGAA R: TAGAAGCATTTGCGGTGG
Hs *ROBO4*	F: CTCGGCTGTCTGTGGCTGTC R: TGTAGGTCCCTTCGTCACTCTT
Hs *DNMT1*	F: CTACCAGGGAGAAGGACAGG R: CTCACAGACGCCACATCG
Hs* TET2*	F: AAGCCCGTGAGAAAGAG R: CCTGTGACCCGAGTGAA
Hs *GAPDH* promoter (−139 to 27)	F: TACTAGCGGTTTTACGGGCG R: TCGAACAGGAGGAGCAGAGAGCGA
Hs *ROBO4* promoter (−183 to −367)	F: AGTAAACATGAGCTGGGATC R: GAAAGGTGGTTGGAGTAAAA
Hs *ROBO4* promoter(−1800 to −2100)	F: TGCCTATTCTTTAGCCTC R: GTTCCCGGAAGGCCAAGG
Hs *ROBO4* promoter(−2400 to −2700)	F: TTTCATCATTACTTTATCCCTCA R: CTGCAGGCTGGAGAGGCTGA
Ms *β-actin*	F: CTGTGCCCATCTACGAGGGCTAT R: TTTGATGTCACGCACGATTTCC
Ms *ROBO4*	F: GTGGAAAGACGGGAAACC R: AATGCGAACAGCCAGAAG
Ms *TET2*	F: TCTACACGAGACAGCCTAC R: GAGATGGTCCTGGTTTG

^a^*Hs* human, *Ms* mouse

^b^*F* forward primer, *R* reverse primer

### Western blot

Total protein was extracted from HRECs or retinal tissues using a Whole Cell Lysis Assay (Wanleibio, Shenyang, China), and the protein concentration was detected using the BCA Protein Assay kit (Wanleibio). Forty micrograms of total protein were separated by 6%, 8%, or 12% SDS-PAGE and were transferred onto polyvinylidene fluoride membranes (Millipore, Bedford, MA, USA) at 80 V for 90 min. After blocking with 5% skimmed milk for 1 h, the membranes were incubated with primary antibodies, anti-ROBO4 (1:1000; 2022-1-AP, Proteintech, Wuhan, China), anti-DNMT1 (1:1000; 24206-1-APm, Proteintech), anti-TET2 (1:1000; A5682, ABclonal, Wuhan, China), anti-ZO-1 (1:500; WL03419, Wanleibio), anti-occludin (1:500; WL01996, Wanleibio), or anti-β-actin (1:1000; WL01845, Wanleibio) overnight at 4 °C. The membranes were then incubated at 37 °C for 45 min with a horseradish peroxidase-conjugated secondary antibody (1:5000; WLA023, Wanleibio) and developed using enhanced chemiluminescence substrate. All bands were quantified using a Gel-Pro Analyzer, and band densities were normalized to β-actin values.

### Chromatin immunoprecipitation

TET2 and SP1 binding to the *ROBO4* promoter was examined by a chromatin immunoprecipitation (ChIP) assay according to the kit (Wanleibio) manufacturer's instructions. DNA and protein in the HRECs were cross-linked using 1% formaldehyde, and the cross-linked samples were degraded to chromatin fragments by sonication. The chromatin fragments were immunoprecipitated with antibodies against TET2 (21207-1-AP, Proteintech) or SP1 (21962-1-AP, Proteintech), and the positive and negative controls were treated with anti-RNA polymerase II antibody and normal rabbit IgG, respectively. The immunoprecipitated complexes were captured using Protein A beads, eluted, and de-crosslinked at 65 °C overnight, followed by purified DNA recovery using a DNA Gel Extraction kit (Wanleibio). TET2/SP1 binding to the *ROBO4* promoter was quantified by RT-qPCR using specific primers (Table 3) [15]. The target values were normalized to the input controls to obtain the fold-change.

### Immunofluorescence

HRECs were seeded on coverslips at approximately 75% confluence. After fixation with 4% paraformaldehyde and permeabilization with 0.1% Triton X-100, cells were incubated with primary antibodies against 5mC (1:100; #28692S, Cell Signaling Technologies [CST], Danvers, MA, USA) or 5hmC (1:100; #51660S, CST) overnight at 4 °C, followed by incubation with Alexa Fluor 555-conjugated secondary antibodies (1:200; Invitrogen, Carlsbad, CA, USA). DAPI was used to visualize the nuclei. Images were captured using a fluorescence microscope (Olympus, DP73, Tokyo, Japan) at 400 × magnification.

### Transendothelial electrical resistance

The monolayer permeability of HRECs was examined by transendothelial electrical resistance (TEER) using a Millicell-electrical resistance system voltmeter (Millipore, Billerica, MA, USA). HRECs were transfected with lentiviral vectors for 48 h and transferred into the top chambers of a 6-well Transwell plate. Cells were incubated in normal medium until they grew to a confluent monolayer. HREC monolayers were treated under normal or hyperglycemic conditions for another 7 days. The resistance of each group of cells was measured. A Transwell plate containing the same medium but without cells was used as the blank control.

### FITC-Dextran transendothelial permeability assay

The permeability of HRECs was measured using a 6.5-mm Transwell chamber with a 0.4-μm-pore polycarbonate membrane insert (Corning Inc., Corning, NY, USA), as previously described [15]. Briefly, each group of HRECs was seeded in the top chamber of the Transwell system at a density of 10^5^ cells/well in 200 μl normal or hyperglycemic medium, while the bottom chamber was filled with 500 μl of the same medium. The cell monolayer normally reached confluence after 3–4 days. For permeability assays, HRECs were washed, 100 μl of 1 mg/ml FITC-dextran (40 kDa; Sigma-Aldrich, St. Louis, MO, USA) was added to the top chamber, and 500 μl phosphate-buffered saline was added to the bottom chamber. After incubation for 30 min in the dark, 100 μl aliquots were obtained from the bottom chamber, and the fluorescence in these samples was measured at an excitation of 490 nm and emission of 520 nm, using a Varioskan Flash (Thermo Fisher Scientific).

### Migration assay

The migratory ability of HRECs was determined using a 6.5-mm diameter Transwell system with an 8.0-μm-pore polycarbonate membrane insert (Corning), as previously described [15]. Approximately 5 × 10^3^ cells from each group were seeded in the top chamber and were cultured in 200 μl serum-free medium, whereas the bottom chambers were filled with 600 μl medium containing 10% FBS. After migration for 24 h, cells in the top chambers were removed with cotton swabs, and the migrated cells were fixed with 4% paraformaldehyde for 15 min and stained with 0.4% crystal violet (Amresco, Solon, OH, USA) for 5 min. Images were captured using an inverted microscope. Cells were counted in five representative fields at 200 × magnification.

### Tube formation assay

The angiogenic ability of HRECs was examined by a tube formation assay, as described previously [15]. HRECs from each group were seeded at a density of 10^4^ cells/well in 100 μl complete medium in 96-well plates coated with 50 μl Matrigel matrix (Corning Inc., Corning, NY, USA). After incubation at 37 °C for 6 h, the tube network was photographed at 200 × magnification. The average number of capillary-like branches and the total branch length in five microscopic fields were quantified using ImageJ software (NIH, Bethesda, MD, USA).

### Immunohistochemistry

Retinal tissues obtained from mice in different experimental groups were fixed with 4% paraformaldehyde and were embedded in paraffin. After deparaffinization and rehydration, 5-μm-thick longitudinal sections were stained with a hematoxylin–eosin staining solution for histological examination. Other paraffin sections were incubated in antigen-retrieval solution and 3% H_2_O_2_ for 15 min, followed by incubation with primary antibodies against 5mC (1:100; #28692S, CST) or 5hmC (1:100; #51660S, CST) overnight at 4 °C, and incubation with horseradish peroxidase-conjugated secondary antibodies (1:500; Thermo Fisher Scientific) for 1 h at 37 °C. The sections were stained with 3,3'-diaminobenzidine and hematoxylin and were then examined and photographed under an Olympus BX53 fluorescence microscope.

### Evans blue assay

The permeability of the blood–retinal barrier in diabetic mice was examined using an Evans blue assay, as reported previously [26]. Evans blue dye (Sigma-Aldrich) was dissolved in 0.9% NaCl (20 mg/ml) and injected into the tail vein at a dose of 45 mg/kg, after the mice were anesthetized. After allowing the dye to circulate for 2 h, the perfused eyes were enucleated and fixed in 4% paraformaldehyde for 2 h, and the retina was isolated from the eyecup under a dissecting microscope. The retinas were transferred to slides, and images were captured using a fluorescent microscope at 40 × magnification. The percentage of avascular area in the retina was quantified using ImageJ software.

### Trypsin digestion of retinas

A trypsin digestion assay was used to analyze the vascular abnormalities of retinal microvessels [27]. Eyes were enucleated and fixed in 4% paraformaldehyde for 48 h. The retinas were isolated from the eyecups, gently agitated in double-distilled H_2_O overnight at room temperature, and then digested in 3% trypsin (Thermo Fisher Scientific) dissolved in 0.1 mol/l Tris buffer (pH 7.8) at 37 °C for approximately 90 min. After repeated washing, the network of vessels was freed from neural tissue by gentle shaking and manipulation under a dissection microscope. Retinal vessels were mounted on slides and allowed to dry completely for hematoxylin–eosin staining. Images were photographed using a microscope at 400 × magnification, and the number of endothelial cells and pericytes were counted in eight representative fields.

### Statistical analysis

All data are reported as mean ± standard deviation. GraphPad Prism 9 software (GraphPad Inc., La Jolla, CA, USA) was used for comparisons between groups, using Student's *t*-test or one-way analysis of variance. A p-value of 0.05 or less was considered statistically significant.

## Results

### The ROBO4 promoter is actively demethylated during the course of diabetes

**Fig. 1:**
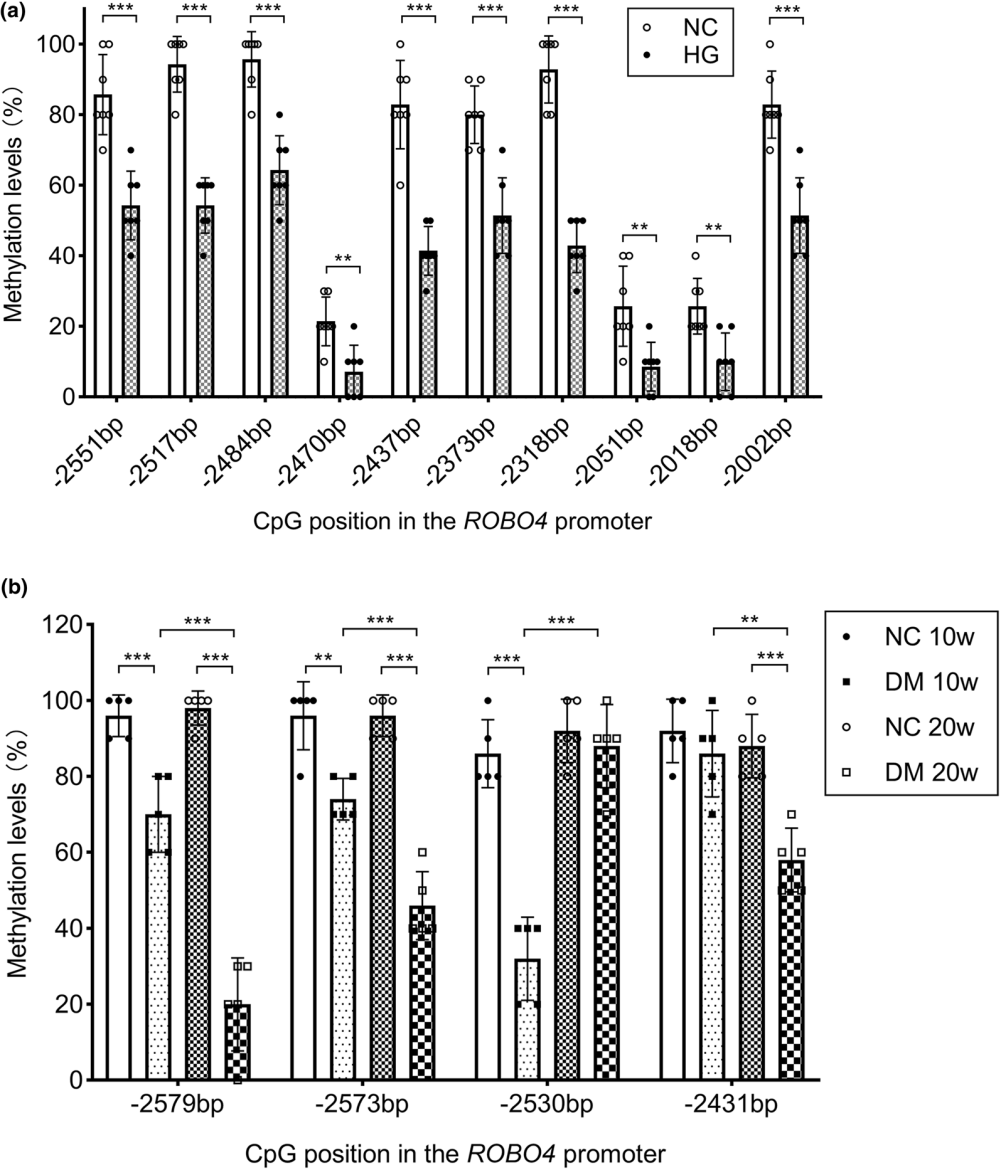
The methylation levels of CpG sites in the 3000-bp promoter sequence upstream of *ROBO4* are reduced in diabetes. **a** The methylation levels of 10 CpG sites in the *ROBO4* promoter in human retinal endothelial cells (HRECs) were detected by bisulfite sequencing polymerase chain reaction (BSP). HRECs were treated with 5.5 mmol/l glucose (NC) or 25 mmol/l glucose (HG) for 7 days. n = 7 (70 clones) for each group. **b** The methylation levels of four CpG sites in the *ROBO4* promoter in mouse retinas were detected by BSP. Non-diabetic mice (NC) and diabetic mice (DM) were fed their respective diets for 10 or 20 weeks. n = 5 (50 clones) for each group. Data represent the mean ± standard deviation. **p < 0.01, ***p < 0.001

We have previously found that the expression of ROBO4 was elevated in diabetic retinopathy [15]. To examine its association with epigenetic alterations of *ROBO4*, the methylation levels of CpG sites in the 3000-bp promoter sequence upstream of *ROBO4* were detected by bisulfite sequencing PCR (BSP) in diabetes, in vitro and in vivo. The methylation region of the *ROBO4* promoter in HRECs contains 10 CpG sites: −2551 bp, −2517 bp, −2484 bp, −2470 bp, −2437 bp, −2373 bp, −2318 bp, −2051 bp, −2018 bp, and −2002 bp. The methylation level of each CpG site was significantly reduced in HRECs cultured under hyperglycemic conditions for 7 days, as compared with that in HRECs cultured under normal conditions (Fig. 1a). The methylation region of the murine *ROBO4* promoter contains four CpG sites: −2579 bp, −2573 bp, −2530 bp, −2431 bp. Compared with normal mice, the methylation level at the 1st and 2nd CpG sites in the retinas of diabetic mice was decreased, and the decrease became more significant with an increased diabetes duration. Methylation of the 3rd CpG site was decreased by 10 weeks of modeling, but returned to normal levels by 20 weeks in diabetic mice. The 4th CpG site showed no difference in methylation levels between diabetic and normal mice at 10 weeks, but the methylation proportion was reduced in diabetic mice at 20 weeks (Fig. 1b).

**Fig. 2:**
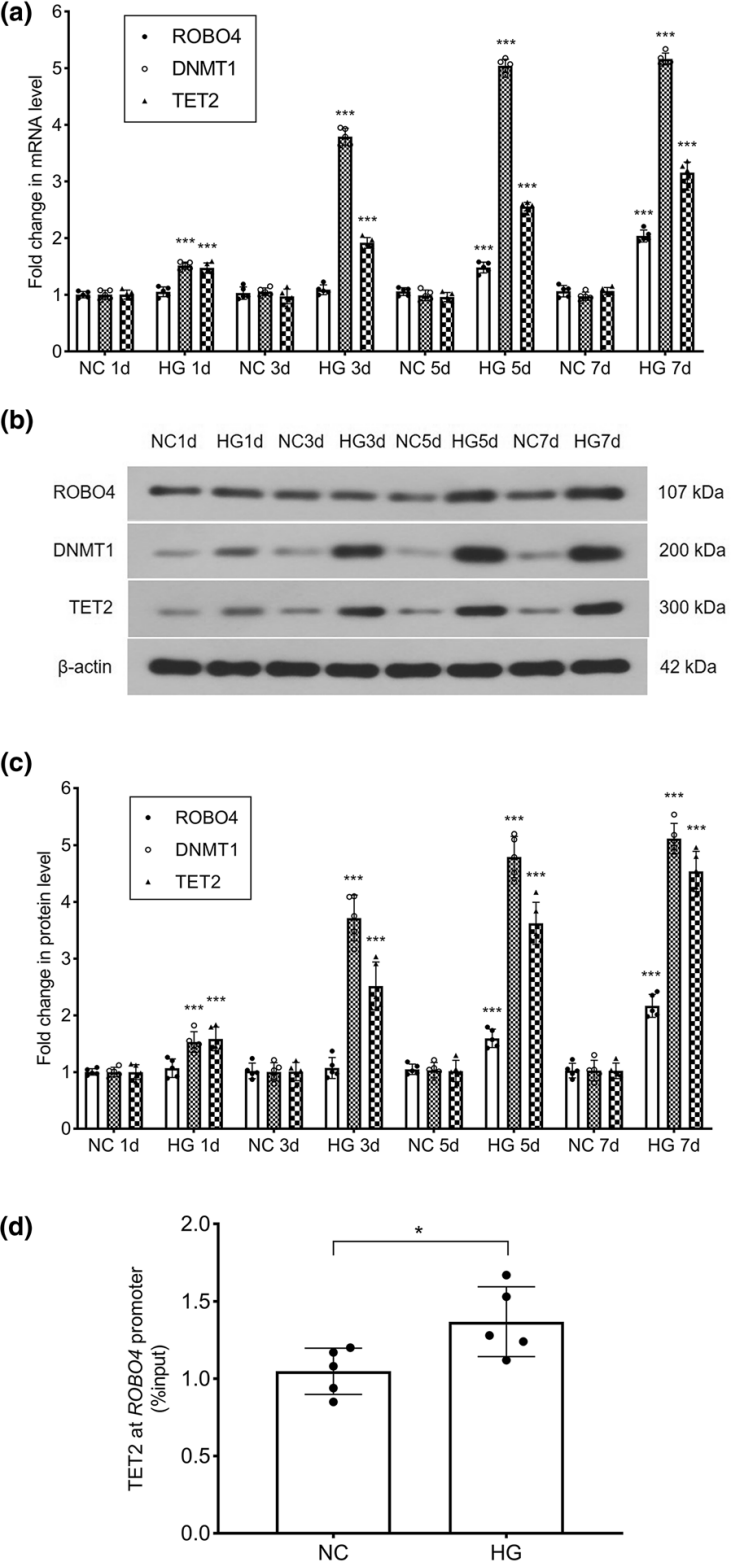
Hyperglycemia upregulates ROBO4, DNMT1, and TET2 transcript and protein levels, and increases the binding of TET2 to the *ROBO4* promoter in human retinal endothelial cells (HRECs). HRECs were treated with 5.5 mmol/l glucose (NC) or 25 mmol/l glucose (HG) for 1, 3, 5 or 7 days. **a** mRNA levels of *ROBO4*, *DNMT1,* and *TET2* were measured by real-time quantitative polymerase chain reaction. *ACTB* was used as the reference gene, and results were analyzed using the delta-delta Ct method. ***p < 0.001 for HG vs NC at the same time. **b**, **c** Protein levels of ROBO4, DNMT1, and TET2 were shown in western blotting analysis and the densitometric analysis was normalized to β-actin expression. ***p < 0.001 for HG vs NC at the same time. **d** Binding of TET2 to the *ROBO4* promoter was determined by the chromatin immunoprecipitation technique. HRECs in the NC and HG groups were incubated for 7 days. Values were normalized to the input controls. *p < 0.05 for HG vs NC. Data represent the mean ± standard deviation, n = 5 per group

To examine the mechanism of demethylation of the *ROBO4* promoter, we detected the mRNA and protein levels of ROBO4, DNMT1, and TET2 in HRECs using RT-qPCR and western blotting analysis. We found that hyperglycemia upregulated the transcript levels of *ROBO4*, *DNMT1* and *TET2*, in an exposure time-dependent manner. These levels increased by 2.04-, 5.16-, and 3.16-fold, respectively, in the hyperglycemic group, at 7 days (Fig. 2a). Changes in ROBO4, DNMT1, and TET2 protein expression levels were similar to those in their respective mRNA levels (Fig. 2b, c). Furthermore, the effect of hyperglycemia on the binding activity of TET2 to the *ROBO4* promoter in HRECs was detected by ChIP. TET2 bound directly to the *ROBO4* promoter, and its binding activity was enhanced in HRECs cultured under hyperglycemic conditions for 7 days, with a value of 1.37% relative to 1.05% for HRECs cultured under normal conditions (p = 0.030) (Fig. 2d).

### TET2 induces active demethylation of ROBO4, enhancing SP1 binding to ROBO4 and decreasing ZO-1 and occludin expression

**Fig. 3:**
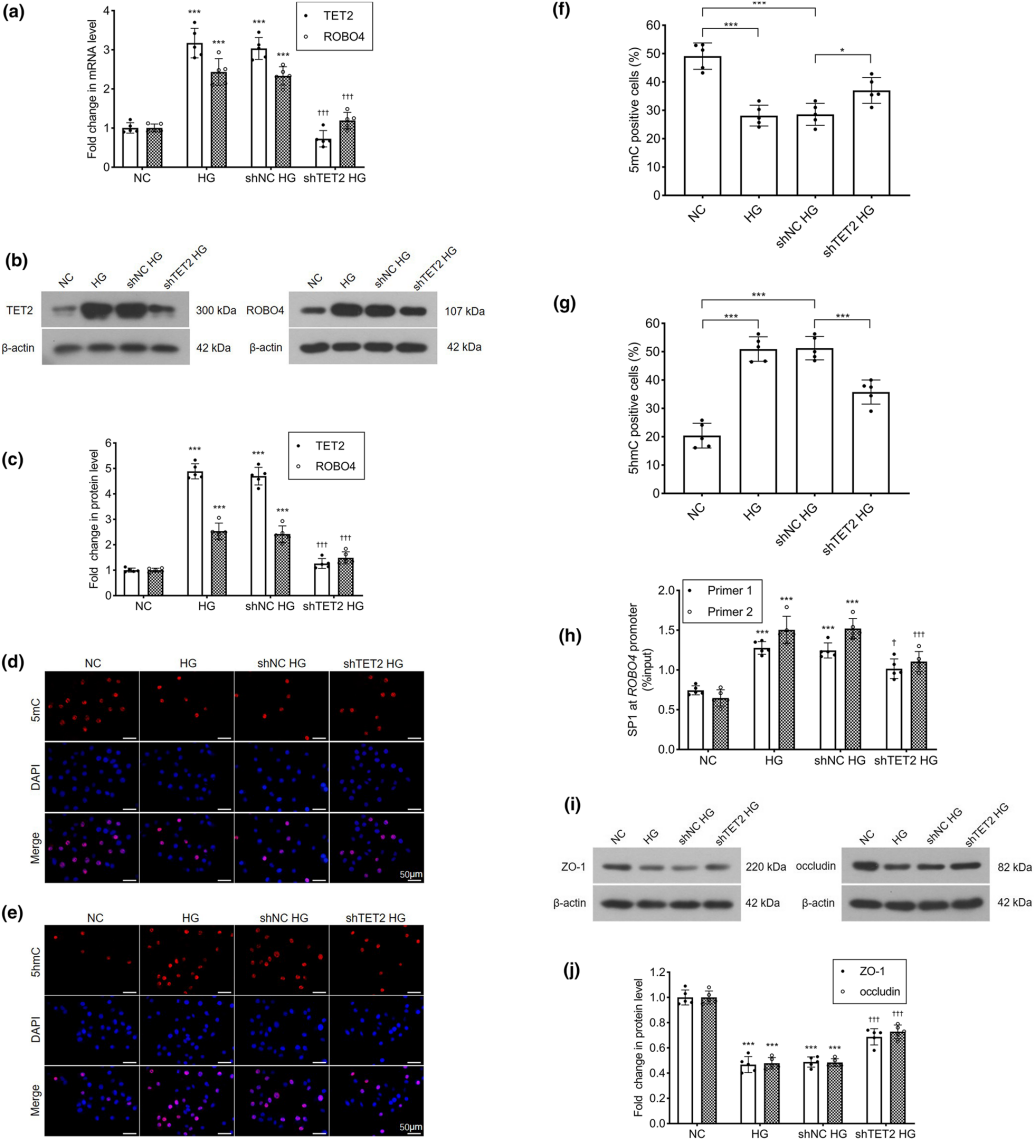
Hyperglycemia induces TET2 to oxidize 5-methylcytosine (5mC) to 5-hydroxymethylcytosine (5hmC), which increases SP1 binding to the *ROBO4* promoter and downregulates ZO-1 and occludin protein expression in human retinal endothelial cells (HRECs). HRECs were cultured under four different experimental conditions: cells cultured in 5.5 mmol/l glucose medium (NC), cells cultured in 25 mmol/l glucose medium for 7 days (HG), cells transfected with control shRNA and incubated in 25 mmol/l glucose medium for 7 days (shNC HG), cells transfected with *TET2* shRNA and incubated in 25 mmol/l glucose medium for 7 days (shTET2 HG). **a** mRNA levels of *TET2* and *ROBO4* were measured by real-time quantitative polymerase chain reaction. *ACTB* was used as the reference gene, and results were analyzed using the delta-delta Ct method. **b**, **c** Protein levels of TET2 and ROBO4 were shown in western blotting analysis and the densitometric analysis was normalized to β-actin expression. **d**, **e** Representative microscopic images of immunofluorescent staining for 5mC and 5hmC (scale bar = 50 μm). **f**, **g** The percentages of 5mC- and 5hmC-positive cells were counted and calculated. *p < 0.05, ***p < 0.001. **h** Binding of SP1 to the *ROBO4* promoter was determined by the chromatin immunoprecipitation technique. Values were normalized to the input controls. **i**, **j** Protein levels of ZO-1 and occludin were shown in western blotting analysis and the densitometric analysis was normalized to β-actin expression. Data represent the mean ± standard deviation, ***p < 0.001 vs NC, †p < 0.05 vs shNC HG, †††p < 0.001 vs shNC HG, n = 5 per group

HRECs were transfected with *TET2* shRNA, to silence TET2, and the mRNA and protein levels of TET2 and ROBO4 were measured by RT-qPCR and western blotting, respectively. The mRNA levels of *TET2* and *ROBO4* were significantly higher in HRECs cultured under hyperglycemic conditions than in the control group. In the *TET2* shRNA transfection group, *TET2* mRNA was successfully knocked-down by 23.98% as compared to the control shRNA transfection. Simultaneously, the *ROBO4* mRNA expression was decreased to 51.11%, approximating the level observed in the normal group (Fig. 3a). A similar trend was observed in the western blotting assay, where *TET2* shRNA transfection downregulated the hyperglycemia-induced elevated ROBO4 and TET2 protein expression levels (Fig. 3b, c). The effect of TET2 on the expression of 5mC and 5hmC was detected by immunofluorescence staining of HRECs. Both 5mC and 5hmC were expressed in the nucleus (Fig. 3d, e). The percentage of 5mC-positive cells was significantly higher than of 5hmC-positive cells in the control group. Under hyperglycemic conditions, 5mC was transformed into 5hmC, and the percentage of 5mC-positive cells decreased and that of 5hmC-positive cells increased. After HRECs were transfected with *TET2* shRNA, the proportion of 5mC oxidized to 5hmC decreased significantly: the percentage of 5mC-positive cells increased and that of 5hmC-positive cells decreased, as compared with the control shRNA transfected (Fig. 3f, g).

Previous studies have shown that there are two binding sites for SP1 in the *ROBO4* promoter in HRECs, and that SP1 binding activity to *ROBO4* was enhanced in hyperglycemia [15]. In this study, HRECs transfected with *TET2* shRNA were cultured under hyperglycemic conditions and the binding activity of SP1 to the *ROBO4* promoter was detected by ChIP. The binding activity of SP1 to *ROBO4* in the *TET2* shRNA transfection group was significantly lower than that in the control shRNA transfection group (Fig. 3h). Expression levels of ZO-1 and occludin, downstream of ROBO4, were detected by western blotting. Compared with the control group, the ZO-1 and occludin protein expression levels were significantly decreased in the hyperglycemia group, and were significantly increased in the *TET2* shRNA transfection group, as compared with the control shRNA transfection group (Fig. 3i, j).

### Inhibition of TET2 expression ameliorates hyperglycemia-induced dysfunction of HRECs

**Fig. 4:**
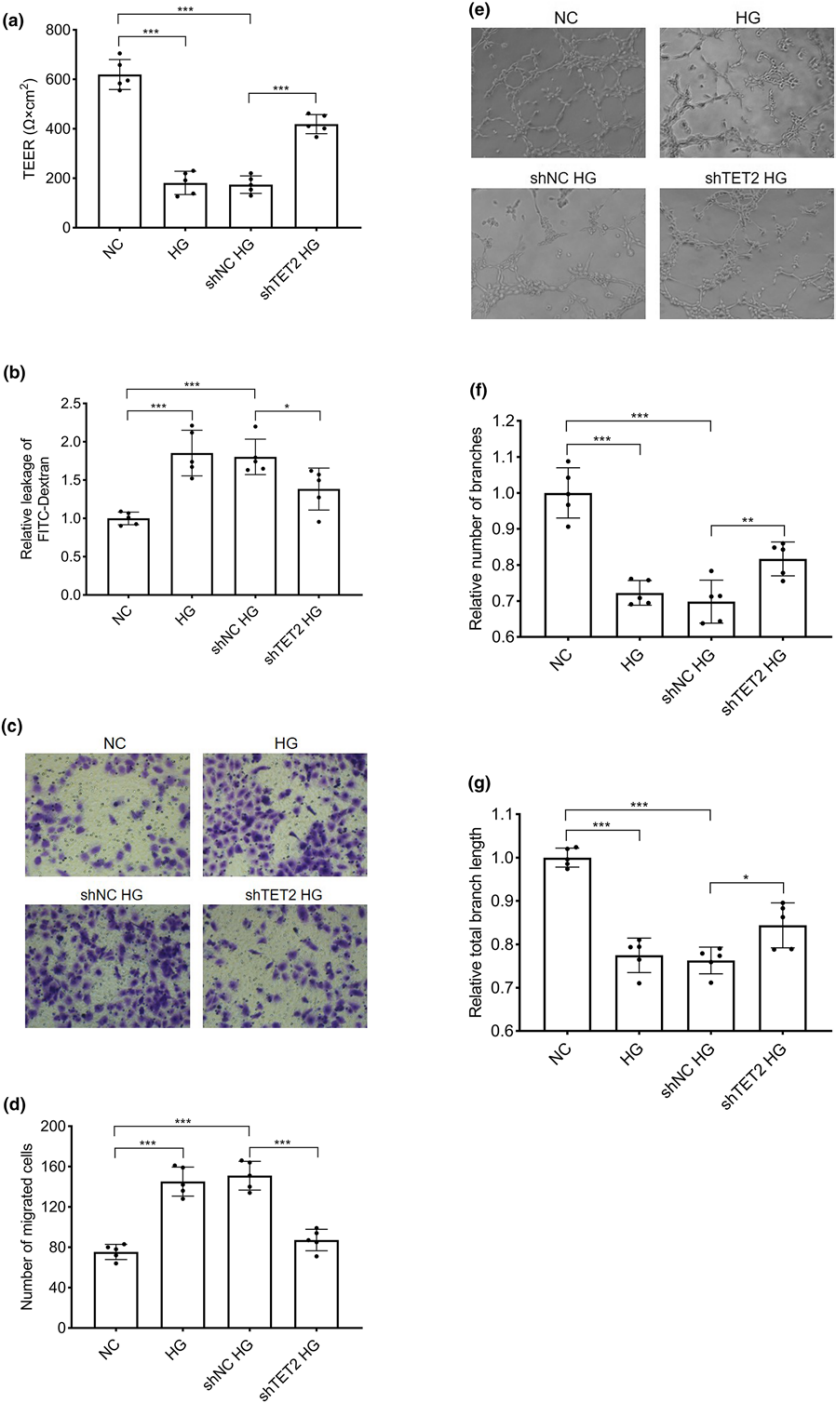
*TET2* shRNA alleviates the hypoglycemia-induced abnormalities of human retinal endothelial cell (HRECs) permeability, migration, and tube formation. HRECs were divided into four experimental groups as described in Fig. 3. **a** The monolayer permeability of HRECs was examined by a transendothelial electrical resistance (TEER) assay. Values were computed by subtracting the resistance (Ω) of the filter alone to each condition and multiplying the result by the total surface of one well (4.67 cm^2^). **b** HREC permeability was analyzed by detecting the leakage of FITC-dextran across cells in monolayer culture. **c** Representative photomicrographs show the migrated cells in Transwell assays. **d** Quantitative analysis of the migrated cells. **e** Representative photomicrographs show Matrigel tube formation of HRECs. **f**, **g** Quantitative analysis of the number of capillary-like branches and the total branch length. Data represent the mean ± standard deviation. *p < 0.05, **p < 0.01, ***p < 0.001, n = 5 per group

The monolayer permeability of HRECs was determined by TEER and FITC-dextran transendothelial assays. As shown in Fig. 4a, compared with the control group, the TEER values in the hyperglycemia group were significantly decreased. When expression of TET2 was inhibited in the *TET2* shRNA transfection group, the TEER was significantly higher than that in the negative transfection group. The FITC-dextran transendothelial assay results were similar to those of the TEER assay (Fig. 4b): *TET2* shRNA effectively inhibited the hyperglycemia-induced increase in HREC monolayer permeability. The role of TET2 in the migratory ability of HRECs was evaluated using a Transwell assay. High glucose levels increased the number of migrated HRECs by 1.93-fold relative to normal glucose levels, whereas *TET2* shRNA transfection markedly decreased the number of migrated cells to 57.75% as compared to the negative transfection group (Fig. 4c, d). Matrigel tube formation in HRECs was also assayed. As shown in Fig. 4e–g, hyperglycemia caused a morphological change in HRECs and a decreased tube network formation as compared to controls. However, *TET2* RNA intervention significantly improved the angiogenic ability of HRECs by increasing the number of capillary-like branches and the total branch length by 1.17-and 1.11-fold respectively, relative to negative transfection cells under hyperglycemic conditions.

### TET2 induces active demethylation of ROBO4, upregulating ROBO4 expression, and downregulating ZO-1 and occludin expression in the retina of diabetic mice

**Fig. 5:**
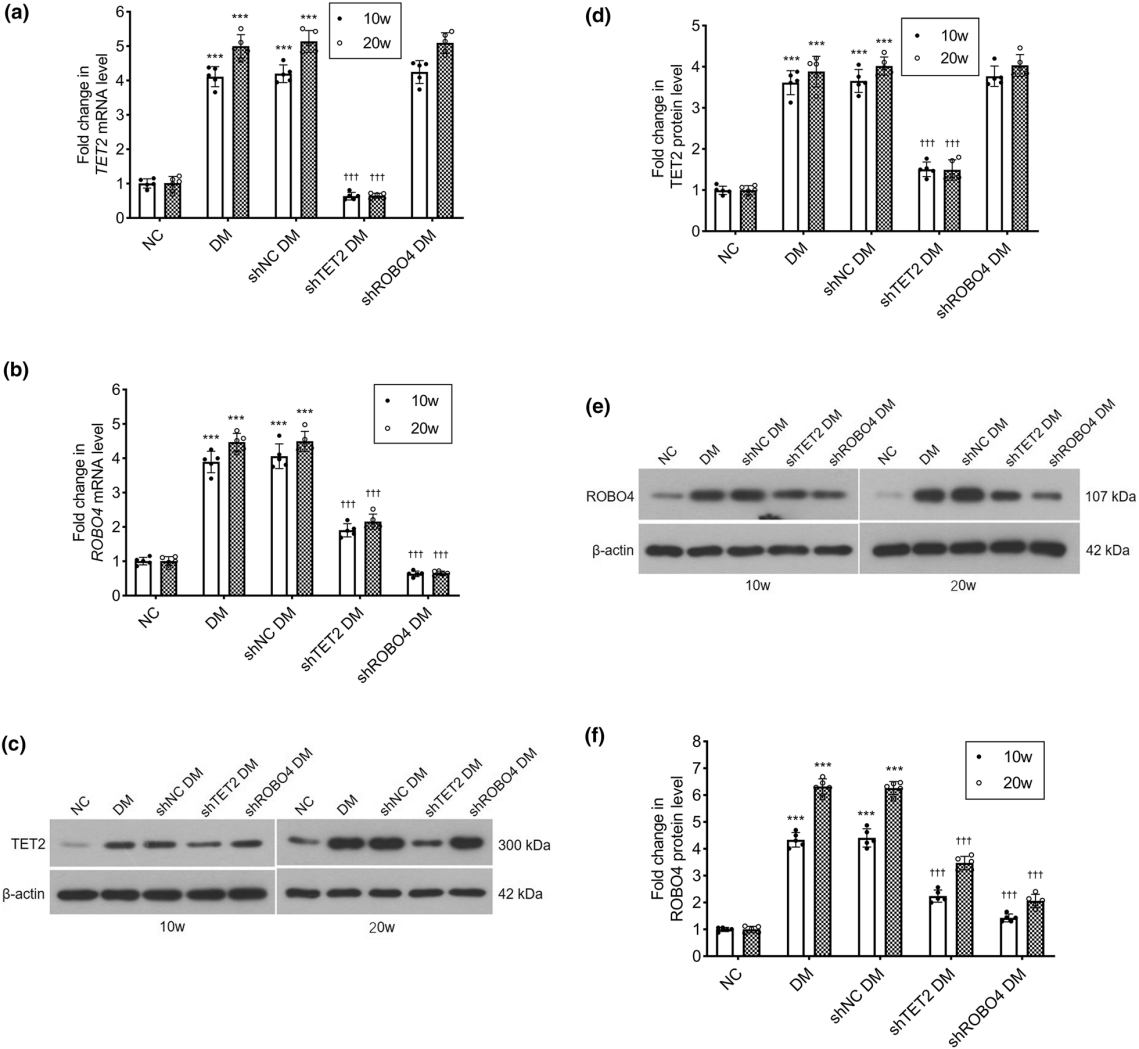
Overexpression of ROBO4 is inhibited by *TET2* shRNA in the retinas of diabetic mice. The mice were divided into five groups: normal mice (NC), diabetic model mice (DM), diabetic mice received intravitreal injection with control shRNA (shNC DM), diabetic mice with intravitreal injection of *TET2* shRNA (shTET2 DM), diabetic mice with intravitreal injection of *ROBO4* shRNA (shROBO4 DM), which were kept for 10 or 20 weeks. **a**, **b** mRNA levels of *TET2* and *ROBO4* were measured by real-time quantitative polymerase chain reaction. *ACTB* was used as the reference gene, and results were analyzed using the delta-delta Ct method. **c**–**f** Protein levels of TET2 and ROBO4 were shown in western blotting analysis, and the densitometric analysis was normalized to β-actin expression. Data represent the mean ± standard deviation. ***p < 0.001 vs NC, †††p < 0.001 vs shNC DM, n = 5 per group

To validate the above pathway in the retina of diabetic mice, adeno-associated virus carrying *TET2* shRNA, *ROBO4* shRNA, or the control shRNA gene was injected into the vitreous cavity of diabetic model mice, and RT-qPCR was used to detect the retinal *TET2* and *ROBO4* transcription levels. *TET2* and *ROBO4* mRNA levels in diabetic mice were significantly higher than those in the control group, but there was no significant difference between diabetic mice and diabetic mice injected with control shRNA, which indicated that the control shRNA had no effect on retinal *TET2* and *ROBO4* transcription. Compared with the control shRNA group, *TET2* mRNA in the *TET2* shRNA group was knocked-down by 15.19% at 10 weeks and by 12.65% at 20 weeks. *ROBO4* mRNA levels were decreased to 46.92% and 48.00%, respectively. In the *ROBO4* shRNA group, *ROBO4* mRNA was inhibited to the level of the control group, but there was no effect on *TET2* transcription (Fig. 5a, b). The expression of TET2 and ROBO4 protein was detected by western blotting: TET2 and ROBO4 protein levels were consistent with the mRNA levels (Fig. 5c–f).

**Fig. 6:**
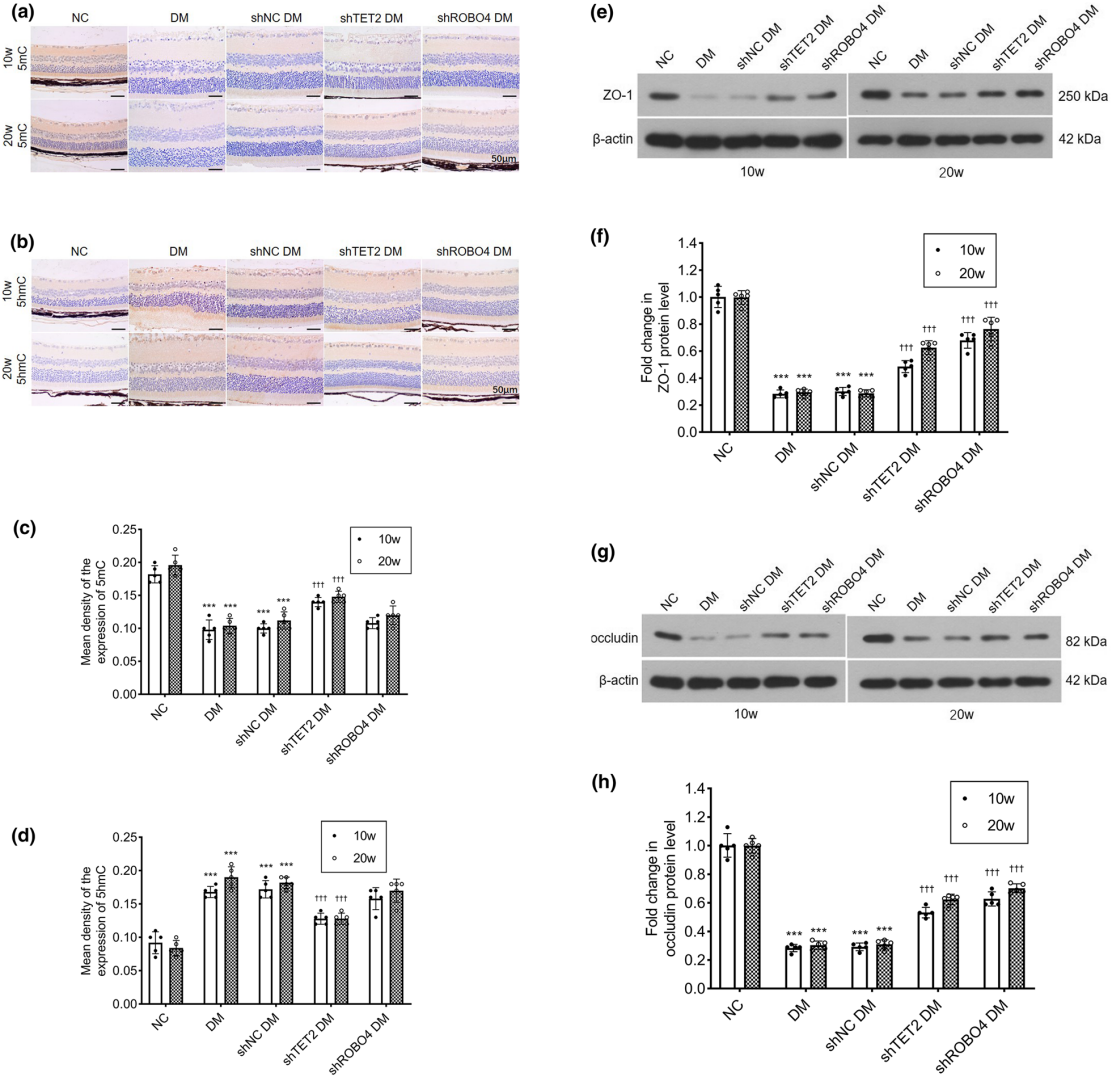
*TET2* shRNA increases the expression of ZO-1 and occludin by inhibiting the oxidation of 5-methylcytosine (5mC) to 5-hydroxymethylcytosine (5hmC) in the retinas of diabetic mice. Mice were divided into five experimental groups as described in Fig. 5. **a**, **b** Representative microscopic images of immunohistochemical staining for 5mC and 5hmC (scale bar = 50 μm). **c**, **d** The mean density (integrated option density/area) of the expression of 5mC and 5hmC were calculated. **e**–**h** Protein levels of ZO-1 and occludin were shown in western blotting analysis, and the densitometric analysis was normalized to β-actin expression. Data represent the mean ± standard deviation, ***p < 0.001 vs NC, †††p < 0.001 vs shNC DM, n = 5 per group

Retinal 5mC and 5hmC levels were detected by immunohistochemical staining. In the control group, 5mC was highly expressed in all retinal layers, whereas that of 5hmC was very low. In diabetic mice, 5mC is oxidized to 5hmC, which results in a significant decrease in 5mC and an increase in 5hmC levels. After intravitreal injection of *TET2* shRNA, the ratio of oxidation of 5mC to 5hmC decreased, the expression of 5mC increased, and the expression of 5hmC decreased, as compared to that in the control shRNA group. However, 5mC and 5hmC levels were not affected by *ROBO4* shRNA injection. Changes in 5mC and 5hmC expression were the same at 10 and 20 weeks (Fig. 6a–d). Western blotting was used to detect the ZO-1 and occludin expression in mouse retinas: ZO-1 and occludin protein levels in diabetic mice and diabetic mice injected with control shRNA were significantly lower than those in the control group. Compared with the control shRNA group, ZO-1 and occludin expression in *TET2* shRNA and *ROBO4* shRNA groups was increased, and more markedly in the latter (Fig. 6e–h).

### Inhibition of TET2 or ROBO4 expression ameliorates abnormal retinal microvasculature in diabetic mice

**Fig. 7:**
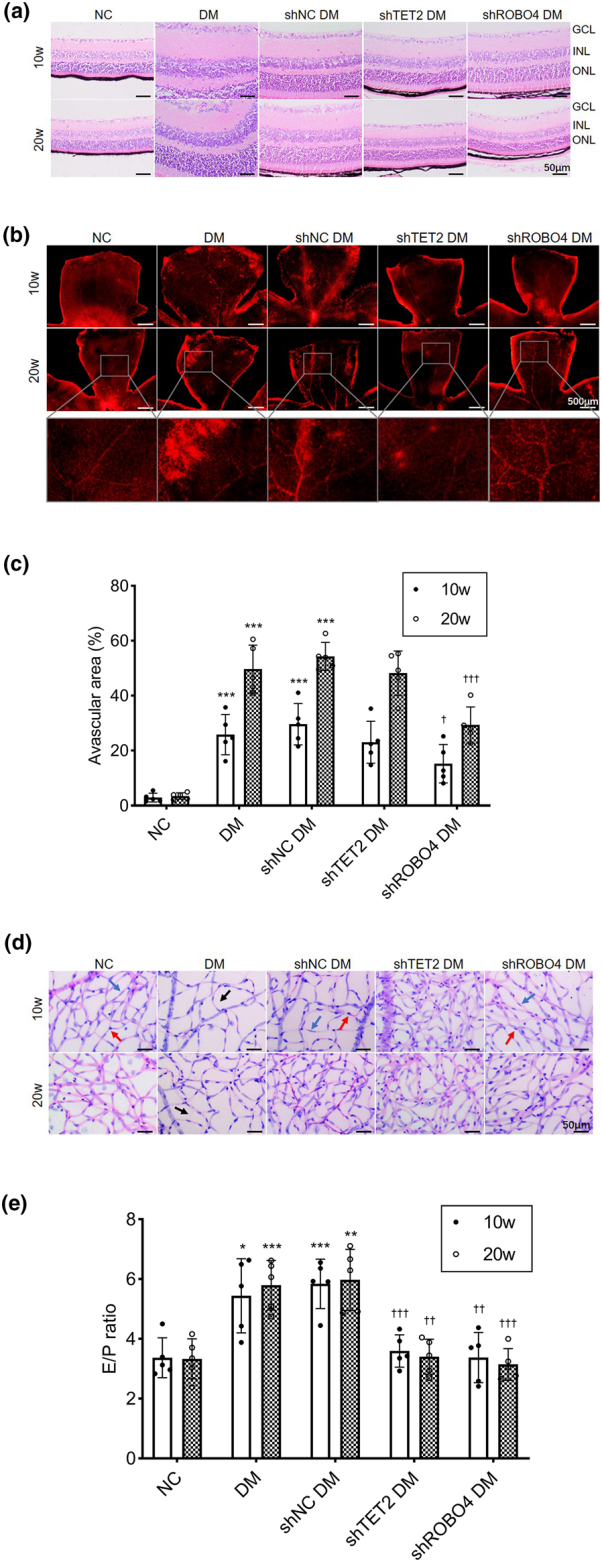
*TET2* shRNA or *ROBO4* shRNA ameliorates the structural and functional abnormalities of retinal microvessels in diabetic mice. Mice were divided into five experimental groups as described in Fig. 5. **a** Representative microscopic images of retinas in diabetic mice stained with hematoxylin and eosin (scale bar = 50 μm). GCL, ganglion cell layer; INL, inner nuclear layer; ONL, outer nuclear layer. **b** Representative photomicrographs of retinal vessels stained with Evans blue (scale bar = 500 μm). **c** Quantitative analysis of the percentage of avascular areas in the retina. **d** Representative photomicrographs of retinal microvessels obtained by retinal trypsin digestion (scale bar = 50 μm). Red arrows indicate pericytes, blue arrows indicate endothelial cells, and black arrows indicate acellular capillaries. **e** Quantitative analysis of the ratio of retinal endothelial cells (E) to pericytes (P). Data represent mean ± standard deviation, *p < 0.05, **p < 0.01, ***p < 0.001 vs NC, †p < 0.05, ††p < 0.01, †††p < 0.001 vs shNC DM, n = 5 per group

We constructed a diabetic mouse model and inhibited retinal TET2 or ROBO4 expression. The retinal tissue sections stained with hematoxylin–eosin showed that normal histological morphology in control mice: the retinal layers were arranged regularly and compactly, and the nuclei were clearly stained. In diabetic mice, at 10 weeks, the retinal tissue layers were irregular and loose, the ganglion cell layer displayed edema and vacuolar degeneration, the inner and outer nuclear layers were arranged disorderly, and the cell density was reduced. These abnormalities were aggravated after 20 weeks of modeling. However, after intravitreal injection of either *TET2* shRNA or *ROBO4* shRNA, the structure of each layer of retinal tissue was regular, the inner and outer nuclear layers were arranged in a relatively orderly manner, and the cell density was increased as compared to that in diabetic mice without these injections (Fig. 7a).

An Evans blue assay was used to assess retinal vascular barrier function in diabetic mice. As shown in Fig. 7b, diabetic mice exhibited more Evans blue leakage areas in the retina than did control mice, and capillary occlusion resulted in the formation of a large avascular zone. In diabetic mice injected with either *TET2* shRNA or *ROBO4* shRNA, leakage of retinal blood vessels was alleviated, and injection of *ROBO4* shRNA significantly reduced the avascular area (Fig. 7c). After 20 weeks of modeling, a large amount of neovascularization was observed in the retina of diabetic mice, but this neovascularization was not observed in diabetic mice injected with either *TET2* shRNA or *ROBO4* shRNA. Retinal microvascular changes in mice were observed by trypsin digestion assay. As shown in Fig. 7d, compared with the control group, the diameter of the retinal capillaries in diabetic mice decreased, the ratio of endothelial cells to pericytes increased, and acellular capillary formation was observed. The pathological changes were aggravated as diabetes was prolonged. However, the retinal vasculopathy improved after inhibiting TET2 or ROBO4 expression. Compared to diabetic mice, the diameter of the retinal vascular network increased, the ratio of endothelial cells to pericytes decreased significantly, the number of acellular capillaries decreased, and improvements were more pronounced in the *ROBO4* shRNA group (Fig. 7e).

## Discussion

ROBO4 is a transmembrane protein expressed in endothelial cells and has been shown to play an important role in developmental and pathological angiogenesis [28]. In mouse models of retinal and choroidal vascular disease, SLIT2 inhibited VEGF-165-induced vascular leakage by activating ROBO4 [10]. ROBO4 could bind to UNC5B to block signaling downstream of VEGF, thereby inhibiting damage to vascular endothelial barrier function [29]. These studies suggested that ROBO4 can maintain vascular integrity. On the other hand, ROBO4 can also promote pathological angiogenesis. In vitro cell experiments showed that SLIT2 could combine with the ROBO1/ROBO4 heterodimer to promote filopodia formation and cell migration [30]. In the blood vessels of some tumors, such as ganglioglioma and bladder cancer, ROBO4 was highly expressed [9, 31], and vaccination against ROBO4 has been shown to inhibit angiogenesis and retard tumor growth [32]. Therefore, ROBO4 is a double-edged sword; it is presumed that the cellular microenvironment and partner proteins determine the proangiogenic or antiangiogenic nature of ROBO4 signaling [33]. In the pathological environment of diabetes mellitus, the expression of ROBO4 is abnormally increased and excessive ROBO4 accelerates the development of DR, as confirmed in our previous studies [12, 13, 15]. To explore the epigenetic mechanism of ROBO4 expression further, we examined the methylation level in the promoter region of *ROBO4*, and our in vivo and in vitro results confirmed that hyperglycemia could induce hypomethylation of most CpG sites in the *ROBO4* promoter, and thereby affect *ROBO4* transcription.

DNA demethylation in animals occurs through a passive or active mechanism [34]. During DNA replication, the new strand produced is not methylated by the maintenance methyltransferase DNMT1 [35], resulting in a progressive dilution of 5mC, termed passive demethylation [36]. Active demethylation involves iterative oxidation of 5mC by TET proteins to form 5hmC, 5-formylcytosine, and 5-carboxylcytosine, followed by base excision repair mediated by thymine DNA glycosylase, which converts 5mC into unmodified cytosine [37–39]. In this study, we examined DNMT1 expression in hyperglycemic HRECs and found that DNMT1 levels increased rather than decreased, indicating that demethylation of the *ROBO4* promoter was not passive. Expression of TET2, which has the greatest effect on retinal capillary cells among TET proteins [18], was further examined. TET2 expression increased significantly under hyperglycemic conditions, and TET2 binding to the *ROBO4* promoter increased, which promoted the transformation of 5mC into 5hmC and induced active demethylation of *ROBO4*, resulting in enhanced binding of SP1 to the *ROBO4* promoter, which promoted ROBO4 transcription and expression. The mechanism of TET2-induced *ROBO4* hypomethylation was also verified in diabetic mouse retinas. Additionally, after inhibiting TET2 expression by shRNA, the oxidation of 5mC to 5hmC and SP1 binding to *ROBO4* decreased, which regulated the abnormal hyperglycemia-induced increase in ROBO4 and returned ROBO4 expression to almost normal levels.

Active hyperglycemia-induced demethylation of *ROBO4* also affected ZO-1 and occludin expression. ZO-1 and occludin are the main components of intercellular tight junctions, which are involved in regulating vascular endothelial permeability [40, 41]. Their expression levels are closely related to the function of important structures, such as the blood–brain [42], glomerular filtration barrier [43] and blood–retinal barriers [44]. In studies of diabetic nephropathy pathogenesis, it is discovered that the disruption in expression and translocation of ZO-1 and occludin in glomerular endothelial cells could be stimulated by reactive oxygen species and RhoA/ROCK pathway [43]. In research on diabetic retinopathy, VEGF [45] and MMP-9 [46] are shown to regulate ZO-1 and occludin in retinal vascular endothelial cells; however, in this study, we discovered a novel signaling pathway that can modulate ZO-1 and occludin. In HRECs cultured under hyperglycemic conditions and in diabetic mouse retinas, along with TET2-induced *ROBO4* hypomethylation and ROBO4 overexpression, ZO-1 and occludin expression was significantly decreased. After inhibition of TET2 or ROBO4 by shRNA, ZO-1 and occludin expression increased, indicating that TET2 could regulate ZO-1 and occludin proteins, mediated by ROBO4, to alter the permeability of retinal microvessels during the course of DR.

The effect of TET2-induced *ROBO4* hypomethylation on the structure and function of retinal vessels was investigated in DR. In vitro, hyperglycemia stimulation increased HREC monolayer permeability, enhanced HREC migratory ability, and reduced HREC tube-formation ability, while TET2 depletion effectively improved these HREC dysfunctions. Our previous studies have also demonstrated that ROBO4 depletion may partially protect against hyperglycemia-induced HREC dysfunction [15]. In vivo experiments showed that, in diabetic mice with a disease course of 10 weeks, retinal structure disorder, retinal blood vessel leakage, pericyte loss, and acellular capillary formation occurred. After 20 weeks of diabetes modeling, these abnormalities were exacerbated with an increase in the retinal avascular area and retinal neovascularization. However, in diabetic mice in which TET2 or ROBO4 expression was inhibited, the retinal vascular system structure and function were effectively protected, and the degree of vascular leakage and neovascularization was significantly reduced. Compared with TET2 depletion, ROBO4 depletion had a more marked protective effect on retinal vessels, which was attributed to the abnormal expression of many downstream factors induced by TET2 depletion, such as ROBO4, MMP-9 [47, 48], transforming growth factor β [49], and interleukin-6 [50], which play different roles in retinal blood vessel regulation. Therefore, the preventive and therapeutic effects of intraretinal ROBO4 inhibition on DR were more accurate.

The treatment of DR remains challenging. The main treatment for patients with mild to moderate NPDR is observation. Intravitreal injection of anti-VEGF medications is the most often utilized therapy when macular edema is present. The advent of anti-VEGF therapy demonstrated remarkable clinical benefits; however, the majority of patients failed to achieve clinically significant visual improvement, and there are limitations such as some cases lacking response to anti-VEGF therapy and short half-life of drugs requiring repeated injection [4], so it is urgent to develop new therapeutic methods. According to a recent clinical study on patients with diabetic macular edema, patients who were insensitive to the anti-VEGF response had significantly higher levels of DNMT1 expression than patients who were sensitive to it [51]. This finding raises the possibility that regulating DNA methylation may be crucial to the complementary treatment of DR. In the present study, we showed that TET-induced *ROBO4* hypomethylation sped up the development of retinal vasculopathy in DR, and it is anticipated that anti-TET2/ROBO4 therapy may be used in conjunction with anti-VEGF therapy or as a supplemental treatment. In order to better target retinal endothelial cells and lengthen the half-life of medications in retinal vessels, we may also combine intravitreal drug injections with nano-biomaterials [52] in future experimental research.

## Conclusion

The present study have demonstrated that hyperglycemia-induced overexpression of TET2 can cause active demethylation of the *ROBO4* promoter, increase ROBO4 expression by activating SP1 binding to the *ROBO4* promoter, and decrease ZO-1 and occludin expression, which leads to retinal vascular leakage and neovascularization. Inhibition of this pathway can effectively delay the development of retinal microangiopathy, thus providing a novel strategy for early intervention in DR.

## Data Availability

The authors confirm that the data supporting the findings of this study are available within the article.

## Abbreviations

5hmC: 5-Hydroxymethylcytosine
5mC: 5-Methylcytosine
BSP: Bisulfite sequencing polymerase chain reaction
ChIP: Chromatin immunoprecipitation
DNMT: DNA methyltransferase
DR: Diabetic retinopathy
HRECs: Human retinal endothelial cells
MEG3: Maternally expressed gene 3
MMP-9: Matrix metalloproteinase-9
NPDR: Non-proliferative diabetic retinopathy
PCR: Polymerase chain reaction
PDR: Proliferative diabetic retinopathy
ROBO4: Roundabout4
RT-qPCR: Real-time quantitative polymerase chain reaction
shRNA: Short hairpin RNA
SP1: Specificity protein 1
TEER: Transendothelial electrical resistance
TET2: Tet methylcytosine dioxygenase 2
VEGF: Vascular endothelial growth factor
ZO-1: Zonula occludens 1
